# Olfactory functioning in adults with Tourette syndrome

**DOI:** 10.1371/journal.pone.0197598

**Published:** 2018-06-06

**Authors:** Martin Kronenbuerger, Patrizia Belenghi, Justus Ilgner, Jessica Freiherr, Thomas Hummel, Irene Neuner

**Affiliations:** 1 Department of Neurology, Johns Hopkins University, Baltimore, Maryland, United States of America; 2 Department of Neurology, RWTH Aachen University, Aachen, Germany; 3 Department of Neurology, University of Greifswald, Greifswald, Germany; 4 Department of Psychiatry, Psychotherapy and Psychosomatics, RWTH Aachen University, Aachen, Germany; 5 Department of Otorhinolaryngology and Plastic Head and Neck Surgery, RWTH Aachen University, Aachen, Germany; 6 Department of Diagnostic and Interventional Neuroradiology, RWTH Aachen University, Aachen, Germany; 7 Department of Otorhinolaryngology, TU Dresden, Dresden, Germany; 8 JARA—Translational Brain Medicine, Aachen, Germany; 9 Institute of Neuroscience and Medicine 4, Forschungszentrum, Jülich, Germany; University of Graz, AUSTRIA

## Abstract

Tourette syndrome is a chronic tic disorder characterized by motor and vocal tics. Comorbidities such as attention deficit hyperactivity disorder and obsessive compulsive disorder can be found. The overlap between neuroanatomical regions and neurotransmitter systems in the olfactory system and the pathophysiology of Tourette syndrome let us hypothesize altered olfactory performance in Tourette syndrome. The main objective of this study was to systematically assess olfactory functioning in subjects with Tourette syndrome and to compare it to healthy controls. We assessed 28 adults with Tourette syndrome (age 33.1±9.4 years, disease duration 23.7±9.7 years) and 28 healthy controls (age 32.9±9.0 years) matched in regard to age, sex, education and smoking habits. The “Sniffin Sticks” test battery was applied to assess odor threshold, discrimination, and identification. Additionally, the combined score of the odor threshold test, the odor discrimination test and the odor identification test of the “Sniffin Sticks” test battery was calculated. Although it was not the primary aim of this study, we assessed whether tics and comorbidity could contribute to olfactory alterations in adults with Tourette syndrome. Therefore, clinical scores were used to assess severity of tics and co-morbidity such as attention deficit hyperactivity disorder, obsessive compulsive disorder, anxiety and depression in subjects with Tourette syndrome. Pathology of the nasal cavities was excluded with rhinoendoscopy. Independent sample t-tests were applied to compare performance in olfactory tests. In the case of statistically significant differences (critical p-value: 0.05), multiple linear regression analysis was carried out to explore whether tic severity, social impairment, co-morbidity or medical treatment had an impact on the differences found. Descriptive values are reported as mean ± standard deviation. Tourette syndrome subjects showed lower combined scores (Tourette syndrome subjects 31.9 ± 5.1 versus healthy controls 35.0 ± 3.1; *p* = 0.007), odor identification scores (Tourette syndrome subjects 12.4 ± 2.0 versus healthy controls 13.7 ± 1.4; *p* = 0.008) and odor discrimination scores (Tourette syndrome subjects 12.1 ± 2.1 versus healthy controls 13.2 ± 1.6; *p* = 0.041) in comparison to healthy subjects, while there was no difference in odor threshold (Tourette syndrome subjects 7.3 ± 2.7 versus healthy controls 8.1 ± 2.2; *p* = 0.22). Seven out of 28 Tourette syndrome subjects (25%) scored in the range of the age- and sex-dependent combined score for hyposmia, while two of 28 healthy controls (7%) had a similar low combined score. None of the participants were found to have functional anosmia. Multiple linear regression analyses suggest that social impairment may a predictor for low combined score and odor identification score in Tourette syndrome subjects (p = 0.003). Compared to healthy controls, altered olfaction in adults with Tourette syndrome was found in this study. Normal odor threshold level but lower scores at tasks involving supra-threshold odor concentrations point towards a central-nervous alteration in the processing of olfactory information in Tourette syndrome.

## Introduction

Tourette Syndrome (TS) is a chronic tic disorder characterized by the presence of fluctuating motor and vocal tics [1–3]. Many people with TS have psychiatric comorbidity and psychopathology such as attention deficit hyperactivity disorder (ADHD), or obsessive compulsive disorder (OCD) [2,3]. TS typically occurs during childhood and puberty, but may persist into adulthood [2,3]. Sensory abnormalities such as the premonitory urge phenomena, enhanced sensory perception, and alleviating maneuvers are reported in TS [1,4,5]. These sensory abnormalities include the sense of smell. More specifically, normal odor threshold but altered subjective sensitivity to odors in adults with TS was reported [1]. Belluscio et al. used the odor threshold test of the Sniffin Sticks to assess olfaction in 19 adults with TS and 19 healthy controls [1]. An odor identification test or an odor discrimination test was not performed. TS subjects reported heightened sensitivity to external stimuli, but there was no difference between TS subjects and controls in the odor threshold test [1]. Findings were thought to result from altered processing in the central central-nervous system in TS[1]. The pattern suggestive for olfactory decline of central nervous origin is (almost) unaffected odor threshold but altered odor identification and discrimination [4,6,7]. To the best of our knowledge, a systematic assessment of olfactory functioning in subjects with TS, including an odor threshold test and tests for odor identification and odor discrimination, has not been reported before.

Neurophysiological, brain imaging and post mortem studies suggest involvement of the cortico-striatal-thalamic-cortical (CSTC) pathways, amygdala, hippocampus, mediodorsal thalamus, cerebellum, insula and cingulate gyrus in the pathophysiology of TS [2,3,8–10]. These structures are also involved in the processing of olfactory information in a complex network [11–20]. Additionally, several neurotransmitter systems are important for signal transmission in the olfactory system and are altered in TS [21]. These include dopamine [3, 5,8,9,21–24], gamma aminobutyric acid [21,25–28] and glutamate [21, 29].

The overlap of neuroanatomical regions and neurotransmitter systems in the olfactory system and the pathophysiology of TS let us hypothesize a link between TS and altered olfactory performance. The primary aim of this exploratory study was to systematically assess olfactory functioning in adults with TS and healthy controls.

## Materials and methods

### Participants

Twenty-eight adult subjects with TS and 28 healthy controls were studied (Table 1). TS subjects were recruited through the TS clinic of the Department of Psychiatry at the RWTH Aachen University and control subjects were recruited via flyers and newspaper advertisements. The diagnosis of TS was confirmed based on established criteria for TS [9,30]. Disease duration was calculated by subtracting the age in years at the time of study by the age in years the criteria for making the diagnosis of TS were met. The years of education were calculated by the years an individual attended school plus the years of training after completing school. Smoking burden was determined by the number of cigarettes (tobacco smoking) used per day at the time of participation in this study. Handedness was assessed by the use of the Edinburgh Handedness Inventory [31].

**Table 1:** Characteristics of participants.

	Tourette syndrome subjects (n = 28)	Healthy controls (n = 28)	p-value*
**Demographics**			
Sex, female / male	7 / 21	7 / 21	
Age	33.1 ± 9.4 years	32.2 ± 8.2 years	>0.05
Education	11.6 ± 1.5 years	11.1 ± 1.7 years	>0.05
Handedness, R / L	25 / 3	26 / 2	
Number of tobacco smokers	14	14	
Smoking burden in smokers	17.6 ± 4.0 cigarettes/day	15.5 ± 4.2 cigarettes/day	>0.05
**Cognitive testing**			
TMT-A	24.3 ± 7.3 sec	23.1 ± 7.6 sec	>0.05
TMT-B	47.6 ± 19.6 sec	44.5 ± 14.8 sec	>0.05
Digital Span forward	9.2 ± 1.9	8.3 ± 1.9	>0.05
Digital Span backward	6.6 ± 2.2	6.7 ± 1.6	>0.05
**Clinical scores**			
YGTSS—motor tics	13.2 ± 4.2		
YGTSS—vocal tics	10.1 ± 5.8		
YGTSS—social impairment	38.9 ± 24.1		
OCS checklist	7.3 ± 9.2		
BSI–depression	58.9 ± 14.1		
BSI–anxiety	64.4 ± 11.0		
WURS–ADHD	32.1 ± 12.6		

Values are means ± standard deviations; YGTSS = Yale Global Tic Severity Scale; OCS = obsessive compulsive symptoms as assessed by the OCS checklist; BSI = Brief Symptom Inventory to assess depression or anxiety; WURS—ADHD = attention deficit hyperactivity disorder as assessed by the German version of the Wender-Utah-Rating-Scale; TMT-A/-B = Trail Making Test–Part A/B; Digital Span test according to Demuth et al.

*p-values as assessed by independent samples t-test, two-tailed.

All participants were interviewed by an experienced physician, board certified for both psychiatry and neurology (I.N.). Healthy controls were matched for age, sex, education and smoking habits to the TS subjects. Healthy controls did not have tics, ADHD, OCD, depression, anxiety or any psychopathology based on clinical interview. Subjects were excluded from participation in this study as healthy controls if they were found to have any of these alterations in clinical interview. Individuals with conditions or illnesses which could impede the sense of smell such as a history of head trauma, chronic or allergic sinusitis, herpetic meningoencephalitis, deformities of the nose, radiation treatment or surgery of the head, or metabolic illnesses such as diabetes mellitus or kidney disease were excluded [4,6,7,14].

Pathology of the nasal cavities was excluded by history and with anterior 30°-angled rhinoendoscopy in all participants [14]. Fourteen subjects with TS took central nervous system active drugs in standard doses (5 selective-serotonin reuptake inhibitor, 1 serotonin and norepinephrine reuptake inhibitor, 4 stimulants, 4 typical and 8 atypical neuroleptics). For the purpose of the data analysis, medication intake was included as a yes (intake of medication) or no (not taking any medication) answer. Nineteen TS subjects were diagnosed with comorbidities such as attention deficit hyperactivity disorder (ADHD), obsessive compulsive disorder (OCD), and psychopathology such as anxiety and/or depression. These subjects may be regarded as having TS plus [30]. In contrast, 9 TS subjects had no psychiatric comorbidity and may be regarded as subject with pure TS [30]. The number of tobacco smokers (n = 14) and their smoking burden (i.e. cigarettes smoked per day) was the same within each group (Table 1).

The ethics committee of the medical faculty at the RWTH Aachen University approved this study (ET 124/07). All participants provided written informed consent prior to testing. The study was conducted according to the principles expressed in the Declaration of Helsinki.

### Clinical assessment

#### Olfactory assessment

The “Sniffin’ Sticks” battery was administered to assess odor threshold, odor discrimination, and odor identification [32,33]. The “Sniffin’ Sticks” are pen-like odor dispensers with a felt-tip (length 14 cm, diameter 1.3 cm). For odor presentation, the cap of an odor pen was removed by the experimenter and the pen’s tip was held approximately 1 cm under both of the subject’s nostrils for approximately 3 seconds. The subjects were tested blindfolded to prevent visual identification of the odorant-containing pens.

Odor thresholds for *n*-butanol were assessed using a three alternative forced choice procedure (3-AFC procedure). Sixteen dilutions were prepared in a geometric series starting from a 4% *n-*butanol solution. Three pens were presented in randomized order, with two containing the solvent and the third containing the odorant. Subjects were asked to identify the odor-containing pen. Reversal of the staircase (i.e., the presentation of the triplet with the next lower odor concentration) was initiated after the odor-containing pen had been correctly identified in two successive trials. In contrast, when subjects gave an incorrect answer, the triplet with the next higher odor concentration was presented and thus, the staircase was reversed. Testing was completed after seven reversals of the staircase. Odor threshold was defined as the mean of the last four turning points out of seven staircase reversals.

In the odor discrimination task, using a 3-AFC procedure triplets of pens were presented in randomized order, with two containing the same and one a different odorant. Subjects had to determine which of the three pens smelled different than the other two pens.

Odor identification was assessed for 16 common odors. A multiple forced choice task was used for this test. Immediately before the odor was presented, the examiner read a list of four response options. The subject was asked to choose one answer option that s/he felt to be correct after the odor had been presented. There was a 30 seconds break between the presentations of the Sniffin’ Sticks pen triplet to avoid habituation or fatigue. Additionally, care was taken that participants had enough time to rest and recover, thereby avoiding movements that could impede testing such as nasal twitches, or head/neck jerks. The maximum score for each of the three olfactory tasks was 16, with lower scores indicating poorer performance.

The composite sum score of odor threshold, discrimination, and identification (TDI) was calculated as a TDI score. We also determined the number of participants with hyposmia or anomia in each group. Based on a multicenter studies involving more than 3000 healthy participants [32,33], hyposmia is defined as a TDI score at the level of the 10th percentile or lower in relation to the subjects’ age and gender. In contrast to hyposmia, functional anosmia is defined as a TDI score of 16 or lower, independent from age and gender [32,33].

Besides testing with the “Sniffin’ Sticks”, all participants were interviewed if they have experienced any alterations in the sense of smell or taste by a simple question (“Do you suffer from any smell or taste problems”) [34].

#### Clinical scores

Although it was not the primary aim of this study, we assessed whether tics and comorbidity could contribute to olfactory alterations in adults TS subjects. Therefore, clinical scores were used to assess severity of tics and comorbidity such as ADHD, OCD, depression or anxiety in TS subjects.

The Yale Global Tic Severity Scale (YGTSS) [35] was applied to evaluate motor and vocal tics as well as social impairment. Obsessive compulsive symptoms (OCS) were assessed via the OCS checklist [36], depression and anxiety by the Brief Symptom Inventory (BSI) [37] and ADHD by the German version of the Wender-Utah-Rating-Scale (WURS) [38]. In contrast to the YTGTSS, the OCS checklist, the BSI and the WURST, the Trail Making Test (TMT) [39] and the Digital Span test [40] were performed to assess executive functioning, attention and working memory in all study participants.

The whole protocol took about 100 minutes, including breaks. The order in which the tests were performed are: 1. consent, 2. collection of demographic information and clinical interview, 3. confirmation of the clinical diagnosis of Tourette Syndrome based on established criteria, 4. olfactory testing and rhinoendoscopy, 5. clinical scores, and 6. cognitive testing.

### Statistical analysis

We used the g*power software to determine sample size [41]. With an alpha of 0.05, an effect size of 0.4, as well as power of 0.8, the total sample size calculated was 34.

Data were investigated using SPSS version 24.0 for Windows (SPSS Inc., Chicago, IL, USA). P-values < 0.05 were considered statistically significant. The test of Kolmogorov-Smirnov indicated that values of the olfactory testing were normally distributed. Descriptive values are reported as mean ± standard deviation. Independent samples t-test (two-tailed) was used to compare performance in olfactory tests between TS subjects and healthy controls. In the case of statistically significant differences between TS subjects and controls, multiple linear regression analysis (“stepwise” method) was carried out in the data from the TS subjects to assess whether disease duration, smoking burden, handedness, medication intake, severity of tics, presence of urge, social impairment, depression, anxiety, ADHD, OCD, or cognitive functioning (performance in trail making test or digit span forward/backward) had an impact upon the olfactory deficit.

Although this study was not powered to discern differences among subgroups (pure TS versus TS plus), exploratory analysis is included in the results. The independent samples t-test, two-tailed, was used for this comparison as well.

## Results

**Fig 1:**
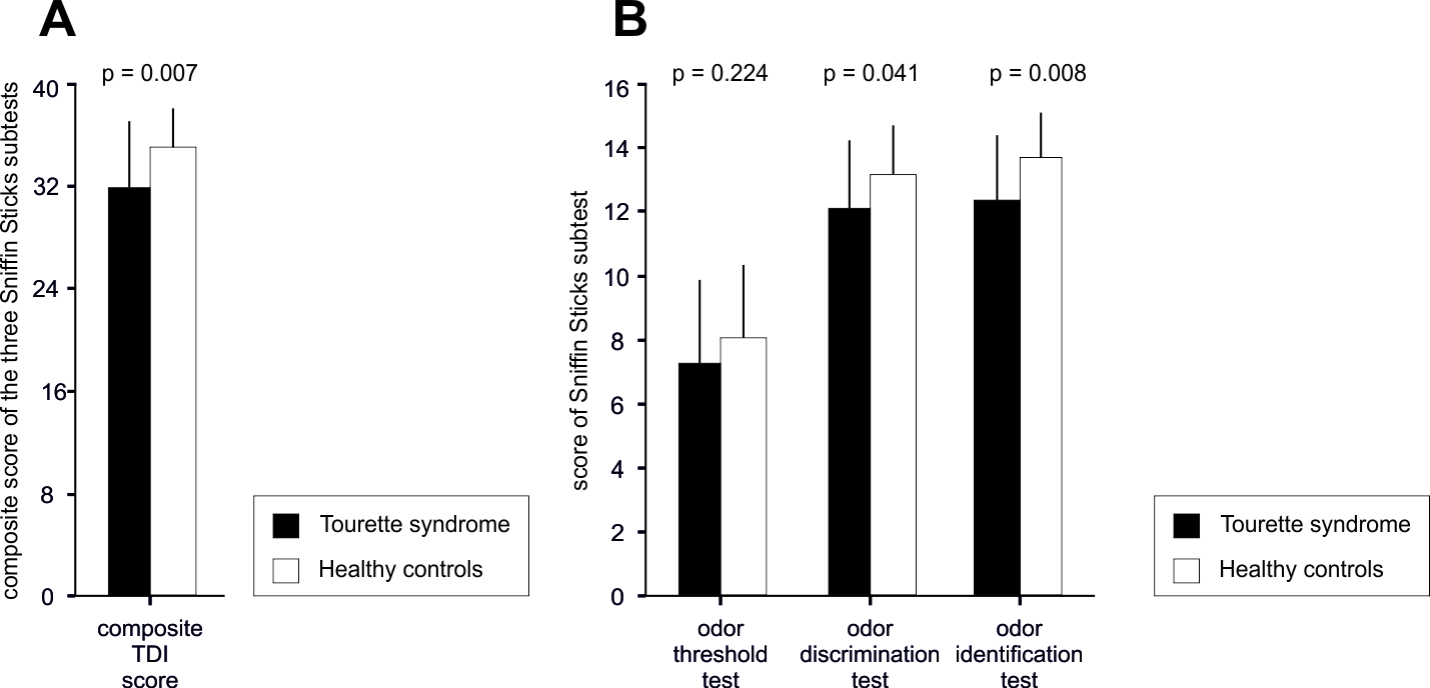
Results from the Sniffin` Sticks test for the two groups examined. (A) Results of the TDI-score, which is the composite sum score of odor threshold test, odor discrimination test, and odor identification test. (B) Results of the odor threshold test, odor discrimination test, and odor identification test. The black bars show the results of the TS subjects, while the white bars show the results from the healthy controls. Each bar indicates the mean and the standard deviation. Lower scores on the Sniffin´Sticks test indicate poorer performance. P-values as assessed by the independent samples t-test, two-tailed.

The group of subjects with TS had lower scores than healthy controls in the composite TDI score (TS subjects 31.9 ± 5.1 versus healthy controls 35.0 ± 3.1; t(54) = 2.80, *p* = 0.007; Fig 1A), the odor identification test (TS subjects 12.4 ± 2.0 versus healthy controls 13.7 ± 1.4; t(54) = 2.77, *p* = 0.008; Fig 1B), and the odor discrimination test (TS subjects 12.1 ± 2.1 versus healthy controls 13.2 ± 1.6; t(54) = 2.09, *p* = 0.041; Fig 1B). There was no difference between groups for the odor threshold test (TS subjects 7.3 ± 2.7 versus healthy controls 8.1 ± 2.2; t(54) = 1.23, *p* = 0.22; Fig 1B).

None of the participants reported altered functioning (decrease or increase) in their sense of smell or taste when they were interviewed.

Multiple linear regressions revealed a significant regression equation for social impairment to predict low performance in the combined score ((F(1,26) = 10.839, p = 0.003), R2 = 0.267, standard coefficient beta = -0.542) and low performance in the odor identification test (F(1,26) = 10.785, p = 0.003, R2 = 0.293, standard coefficient beta = -0.541). All other regression equations did not reach the level of significance.

There was no difference between pure TS subjects and TS plus subjects in any of the olfactory tests (all *p*-values > 0.1). None of the participants were found to have functional anosmia. In terms of hyposmia, seven out of 28 TS subjects (25%) scored in the range of age- and sex-dependent hyposmia TDI score, while two of 28 healthy controls (7%) had a similar low TDI score.

## Discussion

This study sought to systematically investigate the sense of smell in adults with TS. As several brain structures and neurotransmitters are concerned with both the olfactory system and the pathophysiology of TS, we expected altered olfactory performance in TS. In this exploratory study, we found that adult TS subjects had lower TDI scores, lower odor identification scores and lower odor discrimination scores than healthy controls, while there was no difference between the two groups for odor threshold.

Normal olfactory threshold sensitivity in our study confirms findings of a previous study on adult TS patients [1]. Sensory threshold testing in regard to thermal, mechanical [1,42] and olfactory stimuli [1] were found to be normal in adults with TS. No change in odor threshold level (as found in this study and in the study by Bellusio et al. [1]), but significant changes in tasks involving supra-threshold odor concentrations, such as odor identification and odor discrimination (as found in this study), point towards a central-nervous alteration in the processing of olfactory information rather than impaired peripheral detection in TS [4,7,43]. This seems to fit as TS appears to be characterized by central nervous dysfunction in areas which overlap with areas involved in the higher-order processing of olfactory information [5,44].

There are several illnesses and conditions which can impede the brain’s capacity to fulfill certain cognitive tasks such as odor identification or odor discrimination. For example, patients with kidney failure were found to have impaired odor identification [6]. Also, odor identification accuracy declines following sleep deprivation over 24 hours [45]. Thus, one needs to control several factors when studying the sense of smell in order to exclude individuals with illnesses which could impede the sense of smell and to allow study participants to rest before the beginning of the assessment.

Olfactory deficits in adults with TS appeared to be less pronounced than in other movement disorders. For example, 75% of Parkinson’s disease (PD) subjects are hyposmic or anosmic in regard to their gender and age-dependent TDI score [32], while 25% of adult TS subjects had a similar low score. Findings from post mortem studies suggest that patho-morphologic changes in PD also occur in the olfactory system, such as the olfactory bulb and olfactory tract [46]. As a result, olfactory changes are pronounced in Parkinson’s disease [32]. In contrast, post mortem studies in TS have not reported on changes in the olfactory bulb and olfactory tract in TS [47].

Through use of a simple question to inquire about olfactory functioning, we found that none of the participants in this study indicated an alteration of their sense of smell. While olfactory deficits in PD are well documented, up to 80% of people with PD and an impaired sense of smell are unaware of their olfactory deficit when they are interviewed [34]. Interestingly, up to 77% of people in the general population with a measured olfactory dysfunction indicate normal olfaction when they are asked about their sense of smell [48,49] and even 42% of individuals who present to Smell Disorder Clinics are unaware of the extent of their olfactory deficit [50]. Thus, lack of awareness of olfactory deficits appears not to be limited to TS subjects.

The presence of comorbidity such as ADHD and OCD may not impact olfactory alterations in adults with TS as suggested by findings of our study. Interestingly, the sense of smell was been reported to be normal in adults with ADHD or OCD [21,51]. Thus, ADHD and OCD may not to be the critical co-factors for olfactory alterations in TS. We found the severity of motor or vocal tics in TS did not correlate with olfactory impairment found. Likewise, other studies have found altered olfactory performance not to correlate with the severity of motor symptoms of disorders of the central nervous system such as PD, stroke, or ataxia [32,34,52–55]. Multiple linear regression analyses suggest that TS subjects with social impairment due to TS may have a lower combined score and a lower odor identification score. The assessment of a larger number of subjects with TS in future studies may clarify this point. Many studies on olfaction in neurological illnesses were done in a well selected, but relatively small number of participants [32,34,52–55]. Thus, interpretation of findings, especially when it comes to subgroup analysis or analyses of cofactors, has to be made with care.

In regard to drug treatment, statistical analyses in this study suggest that medication in standard drug dosing in TS does not impact performance on olfactory testing. We cannot exclude the possibility that drug treatment in high doses, especially medication which interacts with the dopaminergic system such as neuroleptics, would affect the performance of TS subjects on olfactory tests.

This study has strengths and limitations. It is the first study in which odor identification, discrimination and sensitivity were systematically investigated in adult TS subjects with and without co-morbidity compared to healthy controls matched for age, sex, education and smoking habits. Experimenters were not blinded in this study, as TS subject’s symptoms would have disclosed the diagnosis. However, we used well established and reliable tests to assess olfactory functioning in the participants. Mild deficits in attention can be found in TS, while other cognitive functions such as working memory or verbal fluency are normal [56]. In the subjects assessed in this study, we did not find a group difference in tests of attention. Additionally, performance on the cognitive tests did not correlate with the olfactory alterations found. Therefore, the findings of the present study of adult subjects with TS and compared to healthy controls suggest that the olfactory alterations found are possibly not caused by a cognitive impairment. While an impaired sense of smell in Parkinson’s disease and other neuropsychiatric illnesses are well documented, cognitive assessment in these studies has been limited [32,34,52–55]. In future studies of olfaction in neuropsychiatric illnesses an extended neuropsychological test battery may be applied to assess different cognitive domains in order to correlate cognitive performance with olfactory abilities in greater detail [57]. Additionally, interview instruments such as the olfactory ability questionnaire [58], a visual analogue scale [6] or the Adult Sensory Profile [1,59] may be added to the test battery to increase sensitivity of self-reported olfactory function. This study focused on basic olfactory domains including odor threshold, discrimination and identification assessed with the Sniffin’ Sticks test. Consistent with the established protocol of the Sniffin’ Sticks test, the olfactory stimuli were presented for only a few seconds to avoid habituation [32,33]. There is evidence for impaired habituation to sensory stimuli in TS [1,5]. The assessment of habituation to olfactory stimuli in TS may be the focus of a different study involving olfactory tests other than through the use of the Sniffin’ Sticks. Although having statistical significance for the primary endpoint of this study, the sample size is a limitation of this study. Studying olfactory functioning in a large number of TS subjects may allow subgroup analysis with sufficient power. Finally, we assessed adults with TS. Studying olfactory functioning in children and adolescents with TS may be of interest, as tics are more prevalent in these age groups.

## Conclusion

Compared to healthy controls, altered olfaction in adults with Tourette syndrome was found in this study. Normal odor threshold level but lower scores in tasks involving supra-threshold odor concentrations point towards a central-nervous alteration in the processing of olfactory information in TS. Although the olfactory alterations were small, findings contribute to the understanding of disturbed central processing in TS.

## Supporting information

S1 Table Raw data. (XLSX)
